# TRIM26 facilitates PRV infection through NDP52-mediated autophagic degradation of MAVS

**DOI:** 10.1186/s13567-024-01336-4

**Published:** 2024-07-04

**Authors:** Wu Chengyue, Wang Mengdong, Wang Xiaoquan, Chen Yeping, Li Hao, Sun Liumei, Ren Jianle, Zhang Zhendong

**Affiliations:** 1 School of Biotechnology, Jiangsu University of Science and Technology, Zhenjiang, 212018 Jiangsu China; 2 College of Veterinary Medicine, Shanxi Agricultural University, Jinzhong, 030801 China

**Keywords:** Pseudorabies virus (PRV), TRIM26, MAVS, NDP52, innate immunity, autophagy

## Abstract

Pseudorabies virus (PRV) has evolved multiple strategies to evade host antiviral responses to benefit virus replication and establish persistent infection. Recently, tripartite motif 26 (TRIM26), a TRIM family protein, has been shown to be involved in a broad range of biological processes involved in innate immunity, especially in regulating viral infection. Herein, we found that the expression of TRIM26 was significantly induced after PRV infection. Surprisingly, the overexpression of TRIM26 promoted PRV production, while the depletion of this protein inhibited virus replication, suggesting that TRIM26 could positively regulate PRV infection. Further analysis revealed that TRIM26 negatively regulates the innate immune response by targeting the RIG-I-triggered type I interferon signalling pathway. TRIM26 was physically associated with MAVS independent of viral infection and reduced MAVS expression. Mechanistically, we found that NDP52 interacted with both TRIM26 and MAVS and that TRIM26-induced MAVS degradation was almost entirely blocked in NDP52-knockdown cells, demonstrating that TRIM26 degrades MAVS through NDP52-mediated selective autophagy. Our results reveal a novel mechanism by which PRV escapes host antiviral innate immunity and provide insights into the crosstalk among virus infection, autophagy, and the innate immune response.

**Supplementary Information:**

The online version contains supplementary material available at 10.1186/s13567-024-01336-4.

## Introduction

Pseudorabies virus (PRV), also known as suid herpesvirus 1, is the causative agent of Aujeszky’s disease, which was identified in the United States in 1813 and causes reproductive failure in sows, neurological symptoms in newborn piglets and respiratory disorders in growing pigs, leading to substantial losses in the swine industry worldwide [1]. Moreover, concerns about cross-species transmission of PRV have been raised because variant PRV can directly infect humans and cause endophthalmitis and neurological diseases [2, 3]. The innate immune response provoked by pattern recognition receptors (PRRs) after binding to pathogen-associated molecular patterns (PAMPs) is the first line of defence to protect the host from infection by invading pathogens [4]. To facilitate replication and establish latent viral infection, as a DNA virus, PRV not only evades cGAS-STING- and TLR3-induced antiviral innate immune responses but also suppresses RLR-mediated immune responses [5, 6]. In addition, as a model virus of alpha herpesvirus, PRV has evolved various strategies to modulate other host antiviral immune responses, such as apoptosis, autophagy, and necroptosis [7].

Tripartite motif (TRIM) family proteins are characterized by a RING domain, B-box domains, a coiled-coil domain, and a variable C-terminal region responsible for interactions with different targets [8]. TRIM family proteins have been confirmed to play a very important role in innate immunity, cell proliferation, autophagy, antiviral therapy, and tumour development. TRIM26, a TRIM family protein, was identified as a positive regulator of the RNA virus-triggered innate immune response [9], exerting direct antiviral activity against retroviruses and porcine reproductive and respiratory syndrome virus [10, 11]. Recently, several studies have shown that TRIM26 promotes protein degradation and signal termination by mediating K48-linked ubiquitination and facilitates hepatitis C virus and type 2 herpes simplex virus infection [12, 13]. In addition, the overexpression of TRIM26 impaired the host ability to inhibit the replication of Sendai virus and vesicular stomatitis virus in vivo and in vitro through degradation of polyubiquitinated nuclear IRF3, suggesting a novel and important role for TRIM26 in balancing the innate immune response [14]. However, whether TRIM26 regulates PRV replication remains unknown. In the present study, we explored the interaction between TRIM26 and PRV and found that TRIM26 could facilitate PRV infection by targeting NDP52-mediated autophagic degradation of MAVS. These results reveal a novel mechanism by which PRV escapes host antiviral innate immunity and identify a novel mechanism by which TRIM26 restricts viral infection.

## Materials and methods

### Cell culture, viruses, antibodies, and reagents

HEK293T and PK-15 cells were cultured in DMEM (Gibco, USA) supplemented with 10% foetal bovine serum (FBS, Gibco, USA) and 1% penicillin and streptomycin (P7630, Solarbio) at 37 °C in a humidified atmosphere with 5% CO_2_. PRV strain SD1701 (GenBank accession NO. OR161226) was isolated from Shandong Province in our laboratory. MG132, a proteasome inhibitor, was purchased from Beyotime (S1748). 3-Methyladenine (3-MA, S2767), ammonium chloride (NH_4_Cl, E0151) and Z-VAD-FMK (S7023) were purchased from Selleck Chemicals. Cycloheximide (CHX, A8244), which inhibits eukaryotic protein synthesis, and poly(I:C) (B5551), a Toll-like receptor 3 agonist, were purchased from APExBIO. Sendai virus was obtained from China Agricultural University. Anti-HA (51064-2-AP), anti-Flag (66008-3-Ig), anti-Myc (67447-1-Ig), and anti-GAPDH (60004-1-Ig) antibodies were purchased from Proteintech Group. We acquired an anti-NDP52 antibody (60,732) from Cell Signaling Technology and an anti-MAVS antibody (66911-1-Ig) from Proteintech. The anti-TRIM26 (ab89290) polyclonal antibody was purchased from Abcam, and the anti-EP0 antibody was a gift from the Chinese Academy of Inspection and Quarantine. Human NDP52 siRNA (sc-93738) was obtained from Santa Cruz Biotechnology. Lipo8000 transfection reagent and a Dual Luciferase Reporter Gene Assay Kit (RG027) were purchased from Beyotime. Protease inhibitors were purchased from Roche.

PRV was propagated and titreed in PK-15 cells. For infection, the cells were incubated with virus for 1 h, washed with PBS, and incubated in DMEM supplemented with 5% FBS until the indicated time.

### Generation of TRIM26 knockout cells

To generate TRIM26 knockout cells in HEK293T cells, pX459-sgRNA-puro plasmids targeting the human TRIM26 gene were transfected into 2 × 10^5^ HEK293T cells. Twenty-four hours later, positive (puromycin-resistant) cells were transferred as single clones to a 96-well plate. Candidate clones were screened by PCR, and the PCR products were sequenced to confirm the gene knockout. As a second confirmatory test, the clones were subjected to Western blot analysis using TRIM26 antibodies. As previously described, the HEK293T TRIM26 sgRNA targeting site was designed using Benchling [20]. The sgRNA sequence and PCR sequencing primers used are listed in Additional file 1.

### Transfection, siRNA and dual-luciferase reporter assays

PK-15 or HEK293T cells grown in 24-well plates were transfected with a control empty vector or with a plasmid expressing the indicated protein. Twenty-four hours after transfection, the indicated proteins were detected by Western blotting. The same method was used for siRNA transfection.

HEK293T cells were co-transfected with empty vector or with plasmid as indicated together with pIFN-β-Luc (100 ng), pIRF3-Luc (100 ng), pISRE-Luc (100 ng) or the empty vector (negative control) pGL3 (100 ng) and the Renilla luciferase construct (pRL-TK) (20 ng), which served as an internal control. At 24 h post-transfection, the cells were either left untreated or were treated with 10 ng/mL poly(I:C) or SeV for another 16 h. Luciferase activity was measured using a dual-luciferase reporter assay (DLA) system (Beyotime) and normalized to that of the internal control. The results are expressed as the fold change in luciferase activity relative to that of the mock-treated cells. All assays were performed in triplicate.

### RNA extraction, qRT‒PCR and TCID_50_ assay

The mRNA levels of IFN-β, ISG15, and ISG56 were determined using qRT‒PCR. Total RNA was extracted from the cells using TRIzol reagent (Vazyme, R401-01) according to the manufacturer’s instructions. For RT‒PCR analysis, cDNA was generated with HiScript II Q RT SuperMix for qPCR (+ gDNA wiper) (Vazyme, R223-01), and qRT‒PCR was performed with a ChamQ SYBR qPCR Master Mix (Vazyme, Q331-02) and an ABI QuantStudio 5 Real-time PCR System (Applied Biosystems). GAPDH was used as a reference control, and all of the data are expressed as relative fold changes. The specific primer sequences for the targeting genes are listed in Additional file 1. The virus titre was determined and expressed as the 50% tissue culture infective dose (TCID_50_)/mL as described previously [15].

### Co-immunoprecipitation and western blotting

HEK293T cells were transfected with expression plasmids and incubated for 24 h. The cells were then washed with cold PBS and lysed with cell lysis buffer. For co-IP assays, the cell lysates were centrifuged at 10 000 × *g* for 5 min. The supernatants were incubated with 1 μg of homograft mouse or rabbit antibody and 30 μL of protein A/G-agarose beads (Beyotime, China) at 4 °C for 3 h. The supernatants were collected and incubated with 1 μg of the designated antibody at 4 °C for 12 h, and then each lysate was combined with 30 μL of protein A/G-agarose and incubated at 4 °C for 3 h. The lysates were centrifuged at 2500 × *g* for 5 min to collect the beads, washed five times with cold PBS, added to loading buffer and boiled for 10 min at 100 °C, and then subjected to SDS‒PAGE. The indicated proteins were separated and then transferred to PVDF membranes (Millipore). The membranes were blocked with PBST (PBS with 0.05% Tween-20) containing 5% milk for 2 h at room temperature. After washing, the membranes were incubated with the indicated antibodies for 2 h at room temperature, washed with PBST, and then incubated with HRP-labelled goat anti-rabbit or anti-mouse antibodies for 2 h at room temperature. Finally, the membranes were washed again and reacted with ECL reagents (Thermo Fisher Scientific). Bound proteins were visualized using a Tanon 5200 chemiluminescence imaging system (Biotanon, China).

### Fluorescence microscopy

Cells transfected with plasmids were soaked in 4% formaldehyde and then washed with PBS for 2 min three times. Next, the cells were incubated with blocking buffer for 60 min at room temperature. The diluted primary antibody was added and incubated overnight at 4 °C, after which the cells were rinsed 3 times with PBS for 2 min each.

The diluted secondary antibody was then added and incubated for 1–2 h in the dark at room temperature. After thorough washing with PBS, the cells were counterstained with 2-(4-amidinophenyl)-6-indolecarbamidine dihydrochloride (DAPI) (C1005, Beyotime, Shanghai, China) for 3–5 min and then washed three times with PBS. Finally, the coverslips were mounted using antifade reagent and observed by confocal fluorescence microscopy (U-RFL-T, OLYMPUS, Japan).

### Statistical analysis

All the data were analysed with GraphPad Prism 5.0 using one-way ANOVA or Student’s *t* test. The results are expressed as the mean ± standard deviation (SD). *P* values are indicated using asterisks: *p < 0.05, **p < 0.01, ***p < 0.001; ns, not significant.

## Results

### TRIM26 promotes the replication of PRV

**Figure 1:**
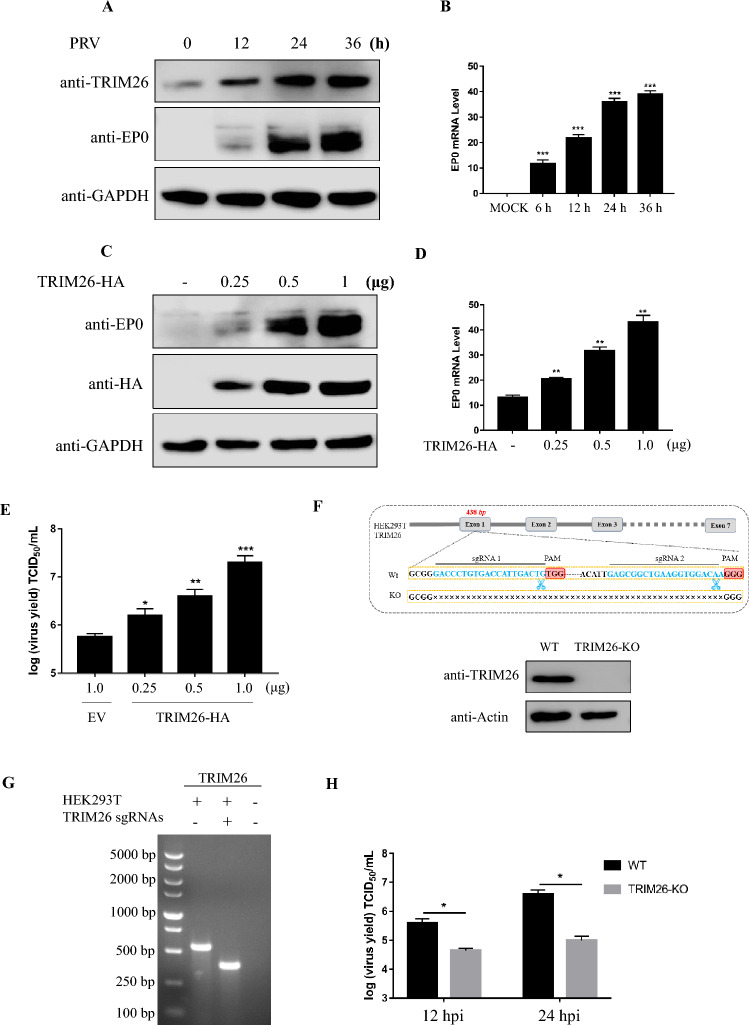
**TRIM26 promotes replication of PRV.** **A** Western blot analysis of TRIM26 protein levels in PK-15 cells after 0, 12, 24 and 36 h of infection with PRV at an MOI of 0.01. GAPDH was used as the protein loading control. **B** mRNA levels of EP0 in PK-15 cells after 0, 12, 24 and 36 h of infection with PRV at an MOI of 0.01. **C** PK-15 cells transfected with TRIM26-HA (0.25 μg, 0.5 μg, or 1 μg) were infected with PRV (MOI = 0.001) at 16 h, and the level of PRV replication was detected by Western blotting at 24 h. GAPDH was used as the protein loading control. **D** and **E** The mRNA levels of EP0 were detected by qPCR (D), and the virus supernatant was collected for the TCID_50_ assay (**E**). **F** and **G** Construction of a TRIM26-deficient cell line using CRISPR/Cas9. TRIM26 deficiency was verified by Western blotting (**F**) and gel electrophoresis (**G**); sequences within TRIM26 were targeted by sgRNA 1 and sgRNA 2 (blue). TGG and GGG (red) are the protospacer adjacent motif (PAM), and “ × ” indicates the knockout sites. **H** TCID_50_ analysis of PRV after 12 and 24 h of infection at an MOI of 0.01. The data are shown as the mean ± SD of three independent experiments; * *p* < 0.1; ** p < 0.01; *** *p* < 0.001.

TRIM26, which contains the RING domain, is theoretically an E3 ubiquitin ligase. Recently, many studies have shown that TRIM26 is involved in the regulation of viral replication and immune signalling pathways. Therefore, we detected the expression of TRIM26 in vitro after PRV infection to investigate the impact of TRIM26 on PRV replication. In fact, TRIM26 expression was significantly upregulated in PRV-infected PK-15 cells, indicating that TRIM26 is associated with PRV infection (Figures 1A and B). Concurrently, the effect of TRIM26 on PRV infection was examined, and the overexpression of TRIM26 resulted in increased expression of EP0 at the protein and mRNA levels (Figures 1C and D) and promoted the replication of PRV in a dose-dependent manner (Figure 1E). To further confirm the effect of TRIM26 on PRV replication, TRIM26 knockout (TRIM26-KO) HEK293T cells were generated using the CRISPR/Cas9 system, and the results were verified by PCR, gel electrophoresis and Western blotting (Figures 1F and G). As shown in Figure 1H, PRV replication was slower in the TRIM26-deficient cells than in the wild-type cells. These results suggest that TRIM26 promotes RRV replication.

### TRIM26 inhibits Type I IFN signalling by affecting the expression of MAVS

**Figure 2:**
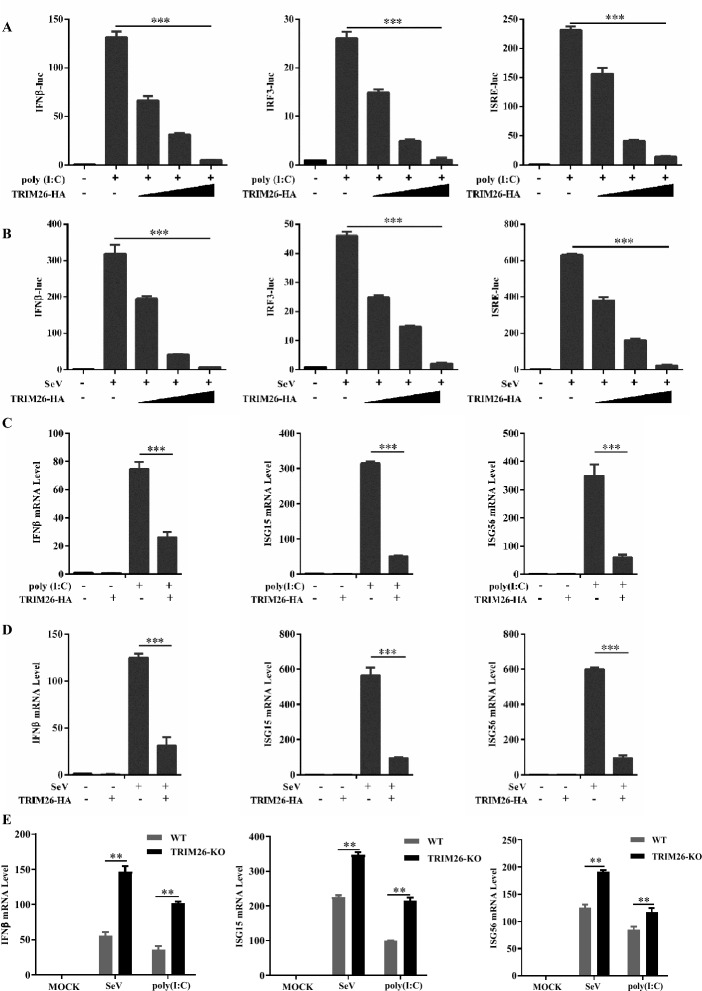
**TRIM26 negatively regulates the Type I IFN signalling pathway.** **A** and **B** TRIM26 inhibits poly(I:C) and SeV-triggered activation of the IFN-β promoter. HEK293T cells were transfected with IFN-β, IRF3 or ISRE reporter plasmids together with TRIM26 expression plasmid or control plasmid and increasing amounts of plasmid expressing TRIM26, and luciferase activity was analysed after transfection with poly(I:C) or infection with SeV. **C** and **D** TRIM26 inhibits poly(I:C) and SeV-triggered transcription of IFN-β and downstream genes. HEK293T cells transfected with TRIM26 expression plasmid or control plasmid were transfected with poly(I:C) or infected with SeV for 12 h, after which the expression of IFN-β, ISG15 and ISG56 was detected via qPCR. **E** TRIM26 deficiency enhances antiviral immunity. WT and TRIM26-KO HEK293T cells were infected with or without poly(I:C) or SeV for 12 h, and total RNA was extracted from the cells and subjected to qPCR analysis for quantitation of IFN-β, ISG15, and ISG56 in HEK293T cells. The data in **A**–**E** are expressed as the mean ± SEM of three independent experiments. **p < 0.01, ***p < 0.001; ns: not significant.

Next, we investigated the mechanism by which TRIM26 enhances PRV replication. Previous studies have shown that TRIM26 targets IRF3, a major transcription factor involved in IFN-β production, leading to IRF3 ubiquitination and subsequent proteasome degradation. To investigate the potential role of TRIM26 in the production of RLR-mediated type I interferons (IFNs), luciferase reporter assays driven by IFN-β, IRF3 and IFN-stimulated response element (ISRE) promoters were performed. The results showed that the overexpression of TRIM26 strongly inhibited the reporter activation induced by poly (I:C) (polyinosinic-polycytidylic acid) transfection and Sendai virus (SeV) infection in a dose-dependent manner (Figures 2A and B). Consistently, the overexpression of TRIM26 in HEK293T cells significantly inhibited the transcription of IFN-β and downstream ISG15 and ISG56, as shown by quantitative PCR (qPCR) (Figures 2C and D). qPCR analysis further revealed that knockout of TRIM26 enhanced the expression of IFN-β, ISG15, and ISG56 triggered by poly (I:C) and Sev in HEK293T cells (Figure 2E). Overall, our data confirmed that TRIM26 could negatively regulate type I IFN production, as described previously.

**Figure 3:**
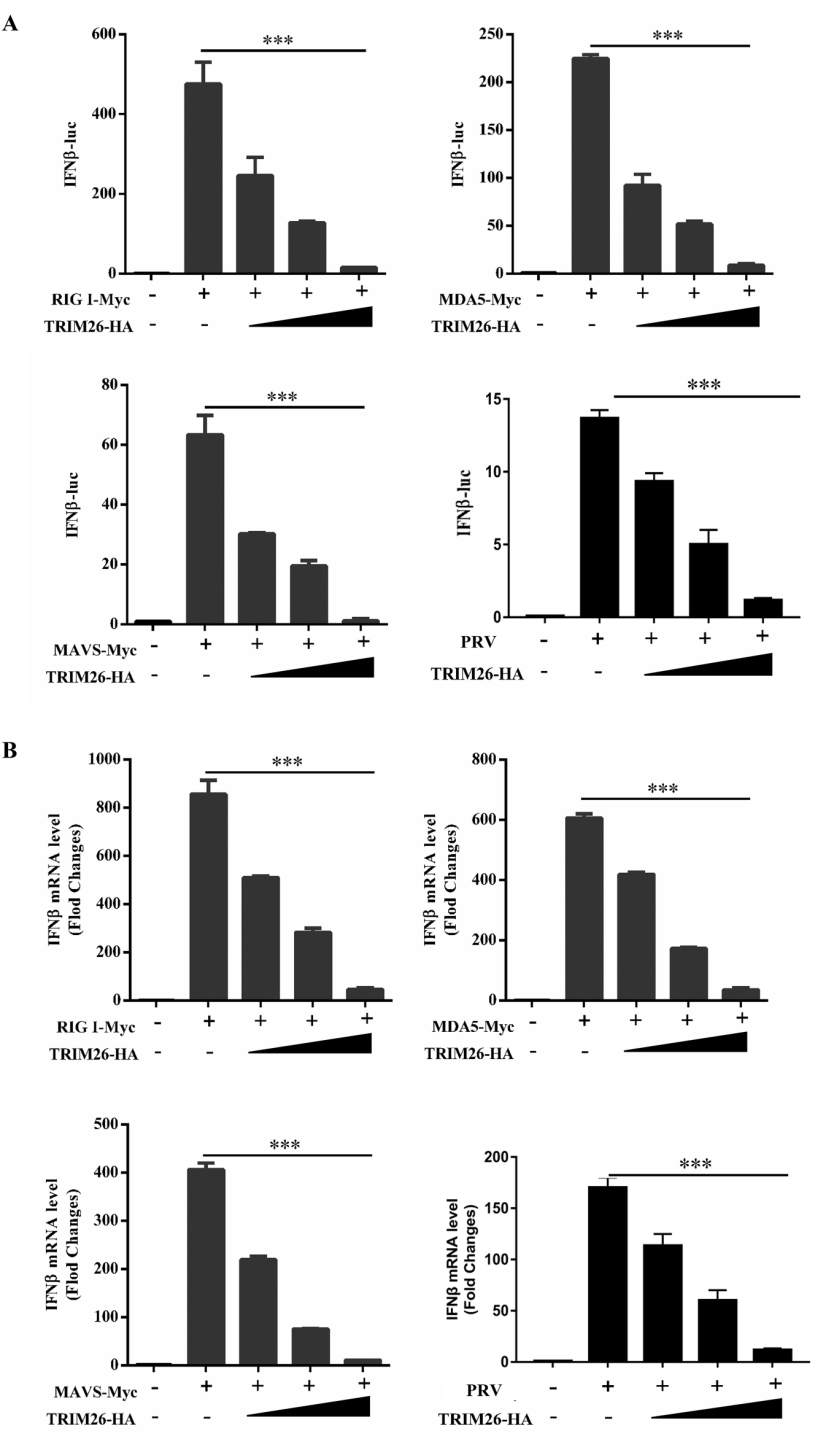
**TRIM26 inhibits RIG-I-mediated type I IFN signalling.** **A** PK-15 cells were transfected with plasmids expressing RIG-I, MDA5 or MAVS or infected with PRV, transfected with the IFN-β reporter plasmid and increasing amounts of the TRIM26 plasmid, and then analysed for luciferase activity. **B** PK-15 cells were transfected with RIG-I, MDA5 or MAVS plasmids or infected with PRV together with increasing amounts of plasmid expressing TRIM26. Total RNA was extracted from cells and subjected to qPCR analysis for quantification of IFN-β. The data are expressed as the mean ± SEM of three independent experiments; **p < 0.01, ***p < 0.001; ns: not significant.

**Figure 4:**
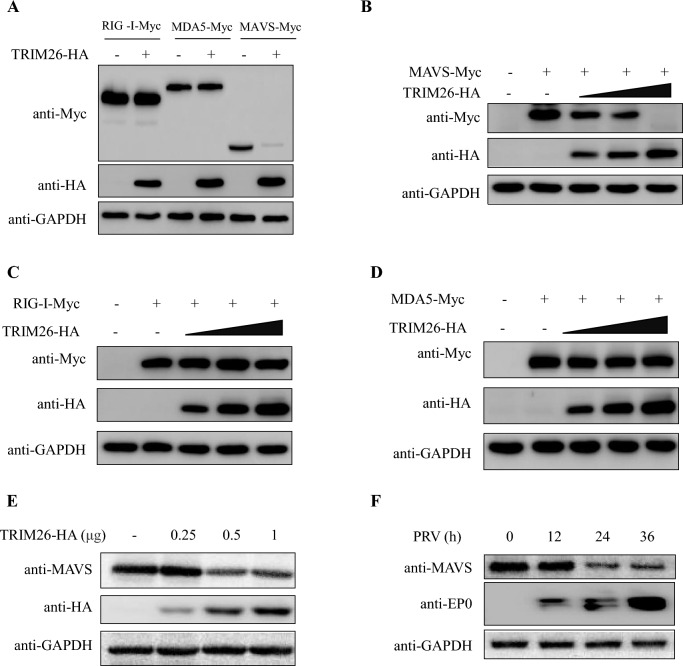
**TRIM26 inhibits Type I IFN signalling by affecting the expression of MAVS.** **A** PK-15 cells were transfected with the indicated plasmids (RIG-I-Myc, MDA5-Myc, or MAVS-Myc) along with control vector or TRIM26-HA expression plasmids. The lysates were analysed by Western blotting with the indicated antibodies. GAPDH was used as the protein loading control. **B**–**D** PK-15 cells were transfected with the indicated plasmids (MAVS-Myc, RIG-I-Myc or MDA5-Myc) along with control vector or increasing amounts of TRIM26-HA expression plasmids. The lysates were analysed by Western blotting with the indicated antibodies. GAPDH was used as the protein loading control. **E** TRIM26-HA inhibited the expression of endogenous MAVS in a dose-dependent manner. **F** PRV infection downregulates endogenous MAVS expression.

To further investigate the inhibitory mechanism of TRIM26 on type I IFN production, we focused on the RLR-mediated signalling pathway. Plasmids encoding RIG-I, MDA5, and MAVS were transfected with the IFN-β promoter in the presence or absence of TRIM26, as determined by a luciferase reporter assay. Overexpression of TRIM26 inhibited IFN-β promoter activation triggered by RIG-I, MDA5, and MAVS stimulation and PRV infection in a dose-dependent manner (Figure 3A), and similar results were obtained by qPCR (Figure 3B). In addition, we detected the protein expression of RIG-I, MDA5, and MAVS; surprisingly, the expression of MAVS was significantly inhibited when the cells were co-transfected with TRIM26 (Figure 4A), and the amount of the MAVS protein was negatively correlated with the amount of the TRIM26 protein (Figure 4B). However, the expression of RIG-I and MDA5 did not change with increasing TRIM26 (Figures 4C and D). In addition, the endogenous expression of MAVS was inhibited after the overexpression of TRIM26 and PRV infection (Figures 4E and F). Collectively, these data demonstrate that TRIM26 suppresses the type I IFN response by targeting MAVS.

### TRIM26 specifically interacts with MAVS

**Figure 5:**
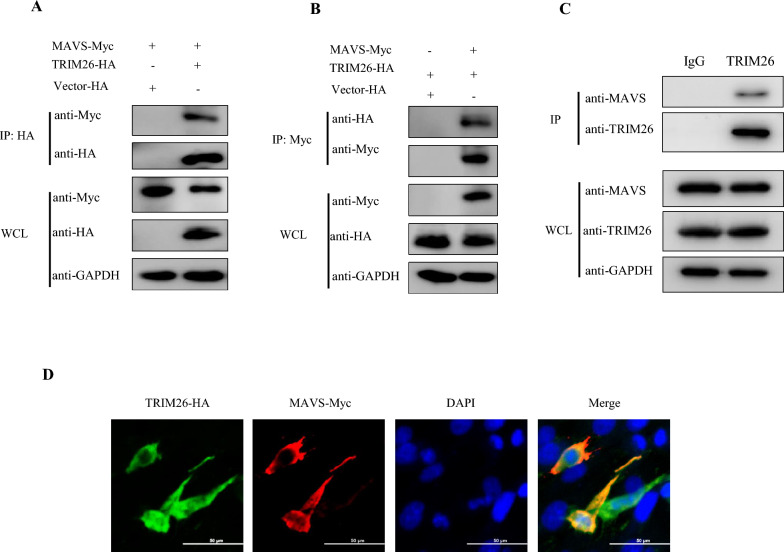
**TRIM26 specifically interacts with MAVS.** **A** and **B** TRIM26 interacts with MAVS. PK-15 cells were transfected with the indicated plasmids for 24 h. Then, coimmunoprecipitation and immunoblotting analyses were performed with the indicated antibodies. GAPDH was used as the protein loading control. **C** Western blot analysis of endogenous MAVS and TRIM26 in immunoprecipitates. **D** TRIM26 colocalized with MAVS in the cell cytoplasm. PK-15 cells were transfected with a TRIM26-HA plasmid for 24 h and then stained with a MAVS-Myc antibody. The nuclei were stained with DAPI. The fluorescence intensity profile of DAPI (blue). TRIM26-HA (green) and MAVS-Myc (red) were measured along the line drawn by ImageJ. Scale bars, 50 μm.

Given that TRIM31 has been found to interact with MAVS and promote MAVS aggregation and activation, it was hypothesized that TRIM26 may regulate innate immunity by interacting with MAVS. Consistent with this supposition, co-IP analysis revealed that Myc-tagged MAVS interacted with HA-tagged TRIM26 (Figures 5A and B). To further determine their relationship, the interaction between the two endogenous proteins was detected by co-IP, and the colocalization of MAVS and TRIM26 was investigated by fluorescence microscopy. The results confirmed the colocalization of TRIM26 and MAVS in the cytoplasm (Figures 5C and D). Taken together, these data suggest that MAVS is a specific target of TRIM26.

### TRIM26 mediates the autophagic degradation of MAVS

**Figure 6:**
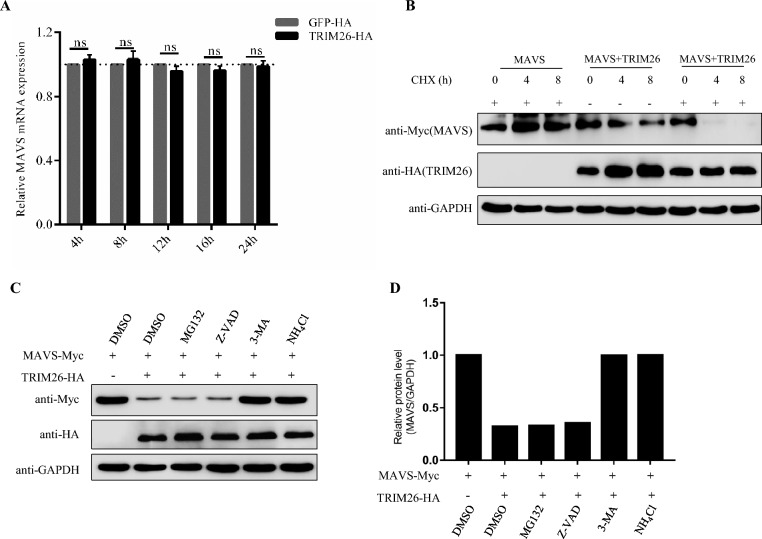
**TRIM26 mediates the autophagic degradation of MAVS.** **A** Overexpression of TRIM26 had no significant effect on the mRNA level of MAVS. The mRNA levels of MAVS in PK-15 cells transfected with TRIM26 were quantitated by qRT‒PCR at the indicated time points post-transfection. The results are representative of three independent experiments. NS, not significant (*p* > 0.05). **B** TRIM26 significantly downregulated the protein level of MAVS. PK-15 cells co-transfected with MAVS-Myc and the control plasmid or with MAVS-Myc and TRIM26-HA were treated with CHX (10 μg/mL) or mock control for 0, 4, or 8 h. Western blotting was used to measure the protein levels of MAVS and TRIM26 at each time point. GAPDH was used as the protein loading control. **C** TRIM26 mediated the degradation of MAVS via autophagy. Western blots showing the levels of MAVS and TRIM26 in PK-15 cells co-transfected with MAVS-Myc and TRIM26-HA or empty plasmid for 16 h and then treated with DMSO, MG132, Z-VAD, 3-MA, or NH_4_Cl for 10 h. GAPDH was used as the protein loading control. **D** The relative intensity of the bands of MAVS to that of GAPDH, as analysed by ImageJ software.

To determine whether the decrease in the MAVS protein level was caused by decreased transcription of this gene, we used qPCR and found that the abundance of the MAVS mRNA did not change with the overexpression of TRIM26, indicating that TRIM26 may promote the degradation of MAVS at the protein level (Figure 6A). As expected, in the presence of TRIM26, MAVS had a significantly shorter half-life, suggesting that TRIM26 specifically disrupted the protein stability of MAVS (Figure 6B). There are two main protein degradation pathways in eukaryotic cells, namely, the ubiquitin‒proteasome pathway and the autophagy‒lysosome pathway. Next, to explore which protein degradation system is responsible for the decrease in the abundance of the MAVS protein, we treated HEK293T cells with the proteasome inhibitor MG-132, the autophagy inhibitor 3-methyladenine (3-MA), the apoptosis inhibitor Z-VAD-FMK, and the lysosome inhibitor ammonium chloride (NH_4_Cl) or DMSO. TRIM26-mediated degradation of MAVS was mainly reversed by treatment with 3-MA and NH_4_Cl but not by treatment with MG132 or Z-VAD-FMK (Figures 6C and D). These results indicated that TRIM26 mediated the degradation of MAVS through the autophagy‒lysosome pathway.

### TRIM26 promotes PRV replication by targeting NDP52-mediated selective autophagic degradation of MAVS

**Figure 7:**
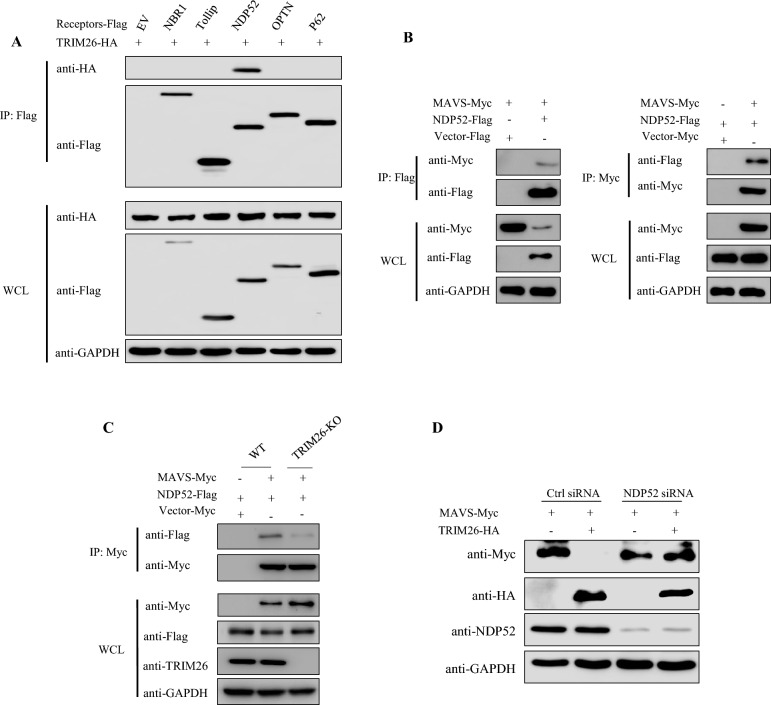
**TRIM26 promotes PRV replication by targeting NDP52-mediated selective autophagic degradation of MAVS.** **A** TRIM26 interacts with NDP52. PK-15 cells were transfected with TRIM26-HA and the indicated Flag-tagged cargo receptors, followed by IP with anti-Flag beads and immunoblot analysis with anti-HA. GAPDH was used as the protein loading control. **B** MAVS interacts with NDP52. PK-15 cells were transfected with the indicated plasmids for 24 h. Then, coimmunoprecipitation and immunoblotting analyses were performed with the indicated antibodies. GAPDH was used as the protein loading control. **C** WT and TRIM26-KO HEK293T cells were transfected with the indicated plasmids for 24 h. Then, coimmunoprecipitation and immunoblotting analyses were performed with the indicated antibodies. GAPDH was used as the protein loading control. **D** Knockdown of NDP52 inhibited the TRIM26-mediated degradation of MAVS. NDP52 siRNAs were transfected with the indicated plasmids for 24 h before Western blot analysis. GAPDH was used as the protein loading control.

Many studies have confirmed that autophagy receptor proteins serve as connectors, linking the targeted substrate with autophagy and mediating the selective autophagy process. Given that TRIM26 is not a cargo receptor, we speculated that TRIM26 might bridge MAVS to cargo receptors for autophagy degradation. Co-IP results showed that TRIM26 interacts specifically with NDP52 but not with other cargo receptors, such as NBR1, Tollip, OPTN, or p62 (Figure 7A). In addition, the use of different antibodies for co-IP indicated that MAVS and NDP52 can interact with each other (Figure 7B). After the deletion of TRIM26, the interaction between MAVS and NDP52 weakened (Figure 7C). Next, after PK-15 cells were transfected with siNDP52, NDP52 silencing restored TRIM26-mediated MAVS degradation (Figure 7D). These results suggested that TRIM26 promotes NDP52-mediated selective autophagic degradation of MAVS.

**Figure 8:**
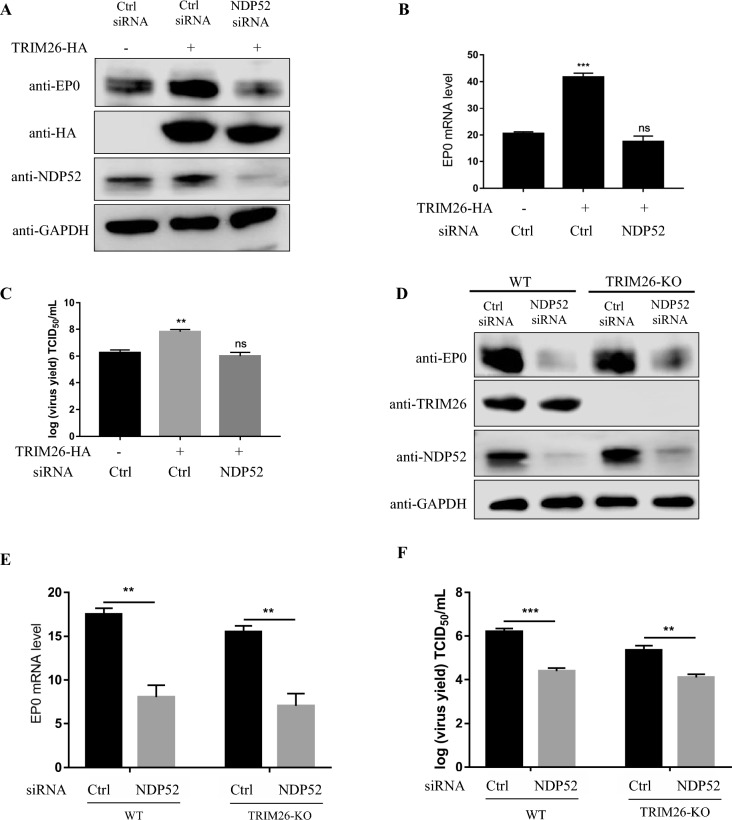
**NDP52 promotes PRV replication.** **A**–**C** PK-15 cells were transfected with NDP52 siRNA for 24 h and then transfected with TRIM26 for 16 h before PRV infection (MOI = 0.001). The expression of HA and NDP52 was determined by Western blotting (**A**), the mRNA levels of EP0 were then detected by qPCR (B), and the virus supernatant was collected for the TCID_50_ assay (**C**). **D**–**F** WT and TRIM26-KO HEK293T cells were transfected with NDP52 siRNA or control siRNA, respectively. Twenty-four hours after transfection, total protein was extracted from HEK293T cells 24 h after infection with PRV (MOI = 0.001). The expression of TRIM26 and NDP52 was determined by Western blotting (**D**), the mRNA levels of EP0 were then detected by qPCR (**E**), and the virus supernatant was collected for the TCID_50_ assay (**F**). The data are shown as the mean ± SD of three independent experiments; *p < 0.1; **p < 0.01; ***p < 0.001.

To determine whether NDP52 mediation is required for TRIM26 to promote PRV replication, we knocked down NDP52 in PK-15 cells, overexpressed TRIM26, and then infected the cells with PRV. We observed a significant reduction in PRV replication (Figures 8A–C), suggesting that TRIM26-mediated promotion of PRV replication requires NDP52. Moreover, after transfecting TRIM26-KO HEK293T cells with siDNP52, we found that the replication of PRV was slightly greater than that in normal cells transfected with siNDP52 (Figures 8D–F), indicating that the replication of PRV was not absolutely dependent on NDP52. Taken together, these results suggest that TRIM26 promotes PRV infection by targeting NDP52-mediated autophagic degradation of MAVS.

## Discussion

Innate immunity acts as the host’s first line of defence against viral invasion, and viruses have evolved various strategies to evade host innate immunity to achieve infection. Toll-like receptors (TLRs), retinoic acid-inducible gene-I (RIG-I)-like receptors (RLRs), NOD-like receptors (NLRs) and intracellular DNA sensors (cGAS, IFI16, etc.) are all well-studied PRRs that detect viral RNA or DNA to trigger cellular signalling cascades, leading to the induction of type I interferons (IFNs), the expression of IFN-stimulated genes (ISGs) and the establishment of an antiviral state [16, 17].

As a DNA virus, the innate immune-induced DNA-sensing pathway plays a key role in controlling PRV infection, but a large number of studies have shown that PRV uses a variety of mechanisms to destroy the host's antiviral response [18]. Yin et al. reported that PRV inhibits type I and type III interferon-induced signalling through proteasomal degradation of JAK1 and Tyk2 [19]. Kong et al. revealed that the PRV tegument protein UL13 recruits RNF5 to inhibit STING-mediated antiviral immunity [5]. Lv et al. demonstrated that UL13 degrades PRDX1 to inhibit IFN production through its kinase activity [20]. Interestingly, studies have revealed that RNA intermediates generated during DNA virus replication and host-derived RNAs induced by DNA viruses can also initiate the RLR-mediated signalling pathway. Recent studies have shown that UL13 of PRV inhibits RLR-mediated IFN-β production by suppressing the expression of RIG-I and MDA5 through the inhibition of NF-κB transcription [6], which is similar to our finding that TRIM26 is recruited by PRV to negatively regulate the RLR signalling pathway. Previous studies have reported that IRF3 can be ubiquitinated and degraded by TRIM26 in the nucleus, suggesting that viral pathogens may induce the expression of TRIM26 to evade innate immunity [14].

In the present study, we found that the expression of TRIM26 was upregulated significantly after PRV infection. Overexpression of TRIM26 inhibited the production of IFN-β, ISG15 and ISG56 triggered by poly (I:C) and SeV; conversely, knockdown of endogenous TRIM26 restored this production. However, Ran et al. reported that TRIM26 physically interacted with TBK1, bridged the TBK1–NEMO interaction, and eventually mediated the activation of innate immune responses [9]. These conflicting reports imply that the role of TRIM26 is versatile and complicated, and the underlying mechanism still needs to be clarified and further studied.

Some alphaherpes viruses have developed effective ways to block RLR-mediated activation of the MAVS–TBK1–IRF3 axis. For example, the VHS protein of HSV-2 decreased the expression of RIG-I and MDA5 [21]. Zhao et al. reported that UL37 of HSV-1 is a protein deamidase that targets RIG-I to block RNA-induced activation [22]. Xing found that HSV-1 US11 binds to RIG-I and MDA-5 and inhibits activation of their downstream signalling [23]. Our previous results revealed that TRIM26 expression increased significantly after PRV infection and that TRIM26 plays a positive role in regulating PRV infection. In the present study, we focused on the mechanism by which TRIM26 inhibits the innate immune response.

To initiate proper immune responses and avoid excessive harmful immune responses, the activities of MAVS, the key component involved in the RIG-I signalling pathway, are tightly and precisely regulated. For instance, the SARS-CoV-2 ORF10 induces mitophagy-mediated MAVS degradation by binding to NIX to suppress the antiviral innate immune response [24]. Senecavirus A-induced glycolysis facilitates virus replication by promoting lactate production, which attenuates the interaction between MAVS and RIG-I [25]. TNKS1 and TNKS2 catalyse the poly-ADP-ribosylation (PARylation) of MAVS at the Glu137 residue, priming it for K48-linked polyubiquitination by the E3 ligase RNF146 and subsequent degradation [26]. The E3 ligase TRIM25 has been shown to interact with ubiquitinated MAVS and be involved in type I interferon production after activation of antiviral RIG-I-like receptors [27]. Interestingly, through IP and confocal microscopy we found that TRIM26 interacted with MAVS and decreased its expression. These findings not only confirmed the negative role of TRIM26 in the innate immune response, as previously described, but also expanded our understanding of how DNA viruses (PRVs) use host proteins to antagonize the RLR-mediated antiviral innate immune response.

Autophagy is a major degradative process mediated by autophagosomes for the breakdown of wrapped contents and was considered to be nonselective when faced with cellular or environmental stresses in early studies [28]. However, an increasing number of studies have indicated that autophagy selectively targets specific substrates through cargo receptors, especially in the context of crosstalk between viruses and host immune responses [29]. For instance, the tegument protein UL21 of alpha-herpesvirus inhibits innate immunity by triggering CGAS degradation through TOLLIP-mediated selective autophagy [30]. Influenza A virus hijacks autophagy to recruit NBR1 to degrade MAVS through autophagosomes, inhibiting the IFN response and promoting virus replication [31]. Porcine reproductive and respiratory syndrome virus utilizes the viral envelope (E) protein to degrade DDX10 via SQSTM1/p62-dependent selective autophagy to antagonize antiviral activity. In addition to virus infection, the iron metabolism-related gene HFE can also bind to MAVS for SQSTM1/p62-mediated MAVS degradation via selective autophagy [32]. Here, the TRIM26-mediated degradation of MAVS was mainly reversed by treatment with 3-MA and NH4Cl but not by treatment with MG132 or Z-VAD-FMK, indicating that the autophagy‒lysosome pathway might be involved in this process. In selective autophagy, p62/SQSTM1 acts as an important adaptor to identify specific organelles and protein aggregates and deliver them to autophagosomes for degradation.

In recent years, considerable progress has been made in understanding the mechanisms underlying selective cargo phagocytosis in mammals, including the identification of ubiquitin-dependent selective autophagy receptors such as p62/SQSTM1, NBR1 (neighbor of BRCA1), OPTN (optineurin), and NDP52 (nuclear dot protein 52 kDa), which can bind both cargo and ubiquitin to initiate pathways leading to autophagy initiation and membrane recruitment [33, 34]. Our data indicated that NDP52 interacted with both TRIM26 and MAVS and that TRIM26-induced MAVS degradation was almost entirely blocked in NDP52-knockdown cells, demonstrating that TRIM26 degrades MAVS by promoting NDP52-mediated selective autophagy. Mounting evidence has shown that ubiquitination is critical for the recognition of cargos in selective autophagy, so whether the degradation observed in our study depends on the RING-finger domain of TRIM26 or the form of polyubiquitin chains linked to NDP52 and MAVS needs to be further studied.

Taken together, the results of our study revealed the additional function of TRIM26 as a negative regulator of DNA virus-induced type I IFN signalling. We propose an important role for TRIM26 in antiviral immune responses, namely, after host infection with PRV, RIG-I-sensing viral transcripts activate RLR-mediated signalling, and TRIM26 is preferentially recruited by PRV and interacts with the selective autophagy receptor NDP52 to recognize ubiquitylated MAVS. NDP52 then delivers MAVS to autophagolysosomes for degradation. TRIM26 downregulates the protein abundance of MAVS, thereby inhibiting TBK1/IRF3 activation and antiviral immunity via negative feedback. Our study identified a novel regulatory mechanism by which TRIM26 evades the host antiviral immune response by triggering MAVS degradation via NDP52-mediated selective autophagy.

### Supplementary Information

**Additional file 1. Sequences of primers used in this study.**

## Data Availability

All of the data analysed during this study are included in this published article. The raw data generated during the current study are available from the corresponding author upon reasonable request.

## Abbreviations

3-MA: 3-methyladenine
ANOVA: analysis of variance
CO-IP: coimmunoprecipitation
IFN: interferon
IRF3: interferon regulatory factor 3
ISG15: interferon-stimulating gene 15
ISG56: interferon-stimulating gene 56
ISRE: IFN-stimulated response element
MAVS: mitochondrial antiviral signalling
MDA5: melanoma differentiation-associated gene 5
NDP52: nuclear dot protein 52 kDa
NLRs: NOD-like receptors
PBS: phosphate buffered saline
PCR: polymerase chain reaction
poly (I:C): polyinosinic-polycytidylic acid
PRV: pseudorabies virus
RIG-I: retinoic-acid-inducible gene I
SD: standard deviation
SeV: Sendai virus
TLRs: toll-like receptors
TRIM26: tripartite motif 26
