# Supplementary material for: TRIM26 facilitates PRV infection through NDP52-mediated autophagic degradation of MAVS
Source: Vet Res. 2024 Jul 4;55:84. doi: 10.1186/s13567-024-01336-4 (PMC11225307; doi:10.1186/s13567-024-01336-4)
Supplement: Supplementary file 1 — Additional file 1. Sequences of primers used in this study. [file 13567_2024_1336_MOESM1_ESM.docx]

**Additional file 1 Primers or sequences were used in this study.**

| Primer | Sequence (5’-3’) |
| --- | --- |
| TRIM26-BamHⅠ-F | CG**GGATCCATGGCCACGT CAGCGCCACT** |
| TRIM26-HA-XhoⅠ-R | GCG**CTCGAG**TCAAGCGTAGTCTGGGACGTCGTATGGGTATCAGGGTCTCAGCAGGAG |
| RIG-I - Kpn I-F | ATA**GGTACC**ATGACCACCGAGCAGCGA |
| RIG-I-Myc- XhoⅠ-R | GCG**CTCGAG**TCACAG ATC CTC TTC AGA GAT GAG TTT CTG CTCTTTGGACATTTCTGCTG |
| MDA5-KpnⅠ-F | GCC**GGTACC**ATGTCGAATGGGTATTC |
| MDA5-Myc-XhoⅠ-R | GCG**CTCGAG**TCACAG ATC CTC TTC AGA GAT GAG TTT CTG CTCATCCTCATCACTAAATA |
| MAVS-KpnⅠ-F | GCC**GGTACC**ATGCCGTTTGCTGAAGA |
| MAVS-Myc-XhoⅠ-R | TAT**CTCGAG**TCACAG ATC CTC TTC AGA GAT GAG TTT CTG CTCGTGCAGACGCCGCCGG |
| qIFN-β-F | GGCTGGAATGAAACCGTCAT |
| qIFN-β-R | TCCAGGATTGTCTCCAGGTCA |
| qISG15-F | GGTGCAAAGCTTCAGAGACC |
| qISG15-R | GTCAGCCAGACCTCATAGGC |
| qISG56-F | AAATGAATGAAGCCCTGGAGTATT |
| qISG56-R | AGGGATCAAGTCCCACAGATTTT |
| qGAPDH-F | ACATGGCCTCCAAGGAGTAAGA |
| qGAPDH-R | GATCGAGTTGGGGCTGTGACT |
| TRIM26-1_CRISPR_F | CACCGGAGCGGCTGAAGGTGGACAA |
| TRIM26-1_CRISPR_R | AAACTTGTCCACCTTCAGCCGCTCC |
| TRIM26-2_CRISPR_F | CACCGGGCCACAGTCAATGGTCACA |
| TRIM26-2_CRISPR_R | AAACTGTGACCATTGACTGTGGCCC |
| TRIM26-JC-F | CTGCTCCATCTGTCTTGATT |
| TRIM26-JC-R | CTCAGGTGGTTCAGGATTTT |
